# Knowledge and practices relating to malaria in a semi-urban area of Cameroon: choices and sources of antimalarials, self-treatment and resistance

**DOI:** 10.4314/pamj.v9i1.71180

**Published:** 2011-05-25

**Authors:** Dickson Shey Nsagha, Anna Longdoh Njunda, Henri Lucien Foumou Kamga, Sarah Mboshi Nsagha, Jules Clement Nguedia Assob, Charles Shey Wiysonge, Earnest Njih Tabah, Alfred Kongnyu Njamnshi

**Affiliations:** 1 Department of Public Health and Hygiene, Medicine Programme, Faculty of Health Sciences, University of Buea, Buea, Cameroon; 2 Department of Epidemiology, Medical Statistics and Environmental Health (Formerly Department of Preventive and Social Medicine), Faculty of Public Health, College of Medicine, University of Ibadan, Ibadan, Nigeria; 3 Department of Medical Laboratory Sciences, Faculty of Health Sciences, University of Buea, Buea, Cameroon; 4 Department of Educational Psychology, Faculty of Education, University of Buea, Buea, Cameroon; 5 Department of Biomedical Sciences, Faculty of Health Sciences, University of Buea, Buea, Cameroon; 6 School of Child and Adolescent Health, University of Cape Town, Cape Town, South Africa; 7 National Programme for Leprosy, Buruli Ulcer & Yaws Control, Ministry of Public Health, Yaoundé, Cameroon; 8 Department of Internal Medicine & Specialties (Dermatology and Neurology), Faculty of Medicine & Biomedical Sciences, University of Yaoundé I, Yaoundé, Cameroon

**Keywords:** Malaria, knowledge, practices, antimalarials, choices, sources, self-medication, resistance, Cameroon

## Abstract

**Introduction:**

Malaria is a major public health problem in Sub-Saharan Africa where it kills a child under the age of five every 30 seconds. In Cameroon, malaria accounts for 40–45% of medical consultations, 57% of hospitalization days and 40% of mortality among children below 5 years. Community knowledge and practices can enhance the fight against this disease. The aim of the study was to make a local analysis of the people's knowledge and practices relating to the choice and source of antimalarials, self-medication, malaria dosage and resistance in order to establish behavioural baseline and epidemiological determinants and their implications for malaria control.

**Methods:**

The design was a community-based cross-sectional study in a semi-urban setting. The survey consisted of 253 volunteer participants (from among 350 contacted) from different socio-demographic backgrounds to whom structured questionnaires were administered. The respondent's consent was sought and gained and subjects who could not read or write or understand English language were communicated to in the local language. The questionnaire was administered by trained interviewers according to the schedule of the respondent. The data was analysed using SPSS.

**Results:**

Antimalarials commonly cited for malaria treatment were chloroquine (26.1%) and nivaquine (14.6%) and analgesics: panadol (23%) and paracetamol (12.3%) including native drugs (6.3%). 141(55.7%) (95% confidence interval (CI): 49.6–61.8%) participants practiced self-medication of malaria. 26.1% participants knew the correct adult malarial dosage for chloroquine or nivaquine. 125(40.4%) (95% CI: 34.4–46.7%) participants got their antimalarials from health centers, 27(10.6%) from shops, 24(9.5%) from hawkers, 23(9.1%) from the open market and 16 (6.3%) from herbalists. 66 (26.1%) (95% CI: 20.7–31.5%) participants knew the correct adult dosage for chloroquine or nivaquine treatment of malaria. 85(33.6%) (95% CI: 27.8–36.6%) participants had correct knowledge of malarial resistance. Of the 85 (33.6%) participants who had correct knowledge of antimalarial drug resistance, 52(20.6%) ascribed antimalarial drug resistance to continuous fever for a long time during treatment, 15 (5.9%) to serious fever during treatment and 18 (7.1%) when chloroquine does not stop fever. 23(27.1%) participants with correct knowledge of malarial resistance were in the 31–35 age group bracket compared with other age groups (P=0.1). There was a significant difference in correct knowledge of malarial resistance and participant's profession (p=0.0).

**Conclusion:**

Malaria self-medication is common in Ndu but knowledge of antimalarial drug resistance is poor. Improvement in the self-treatment of malaria could be attained by providing clear information on choices of drugs for malaria treatment. Proper health information on the rational use of ant-malarial drugs must be provided in an appropriate manner to all groups of people in the society including village health workers, women associations, churches, school children, “Mngwah” opinion leaders, herbalists, health workers and chemists. Self-medication should be improved upon by giving correct information on the dosage of malaria treatment on radio, television, posters and newspapers because banning it will push many people to use it in hiding.

## Introduction

Malaria is the major cause of morbidity and mortality in the tropics [1]. Antimalarial drug resistance, developing from two major factors: prescribing behaviour of health workers and self-treatment of the community is a hurdle to the control of this disease. In the 1990s, the WHO recommended that in areas or seasons of high malaria endemicity, malaria treatment should be given to all patients with fever or a history of fever [2]. The current malaria treatment guideline by WHO [3] stipulates that prompt parasitological confirmation by microscopy or alternatively by RDTs is recommended in all patients suspected of malaria before treatment is started; treatment solely on the basis of clinical suspicion should only be considered when a parasitological diagnosis is not possible. Cameroon adopted the current WHO guideline for malaria treatment and changed from chloroquine or amodiaquine monotherapy to artemisinin-based combination therapy (AS/AQ) in 2005 [4, 5]. Although presumptive treatment of febrile illnesses with antimalarials is part of the national health policy in most African countries, it results in the unnecessary prescription of antimalarials [6]. These treatment policies are common in Cameroon where laboratories are sparsely equipped and where laboratory services are not readily available especially in the peripheral areas. In Cameroon, malaria accounts for 40–45% of medical consultations, 57% of hospitalization days and 40% of mortality among children below 5 years of age [7]. Proper health education of village mothers has been proven to reduce under-five malaria morbidity and mortality significantly in Cameroon [8]. In a study conducted among 1197 health service users in northern Cameroon, only 1% identified mosquitoes as a source of transmission [9]. Contrary to Cameroonians who have poor knowledge of malaria determinants [9], French nationals living in Cameroon are well informed of malaria [10]. Deficient knowledge on malaria treatment guidelines and the irrational use of antimalarials [4] has been reported in Cameroon. There is also evidence of major differences in malaria treatment, prophylaxis and diagnosis in Cameroon [11].

Inadequacy of resources and trained personnel in health care, necessitate the purchase of antimalarials over the counter in many African countries [12]. Self-medication as a means of malaria treatment is common in Cameroon [13]. Fifty six percent women declared that they had taken either chloroquine or amodiaquine during pregnancy because they thought they had malaria [13]. In another separate study in Cameroon [14], most (56.8%) participants admitted that they used drugs for the prevention of malaria, most (95%) of these using chloroquine. Preventing the foci of resistant falciparium malaria from widening requires the rational use of antimalarials [15].

The emergence of chloroquine resistance malaria was first documented in south-western Cameroon (an area hyperendemic for malaria) in 1985 [16]. In 1995 [13], a 10% antimalarial drug resistance to chloroquine was reported from Southern Cameroon. More than 50% chloroquine resistance of *Plasmodium falcipariu*. was reported from the same area in 1997 [14]. Although such resistance has spread rapidly throughout the country, the amodiaquine-artesunate combination is the drug of choice for malaria treatment in Cameroon nowadays [7].

Tremendous efforts towards malaria control in Cameroon have not been cost effective [17–19] resulting in difficulties of eradication of the malaria vectors [20]. Control strategies require public support and the extent of people's cooperation can determine the success or failure of the entire campaign against malaria [21]. This support entails an assessment of the people's knowledge and practices relating to the choice and sources of antimalarials, self-treatment, dosage and resistance, hence this study was conducted.

## Methods

### Study Area

Ndu is a small semi-urban community with a population of slightly over twenty thousand inhabitants located at latitude 6° 24′ 0N, longitude 10° 46′ 0E, and at an altitude of 1827 meters above sea level [22] in the North West Region of Cameroon. It is the highest point in Donga-Mantung division and is always very cold. Ndu is the headquarters of Ndu subdivision in the Wimbum tribe. The Wimbum tribe constitutes the main inhabitants and their dialect is called “Limbum”. Other major tribes in Ndu include Nso, Hausa, Fulani, Yamba and Igbo (Nigerians). It is the central commercial point and acts as one of the main exit and entry routes to Nigeria. Ndu is situated about 160km from the regional capital, Bamenda. Neighbouring villages of Wowo, Mbipgo, Njillah and Kakar where the tea plantation is located were also included in the study.

There is a District Hospital in Ndu and the Baptist Health Centre and the “Ndu Tea Estate” Health Centre. There are two main hospitals in Banso and one in Nkambe, each about an hour′s drive from Ndu. Patent medicine stores are common in this locality and antimalarials are dispensed on daily basis and weekly market days by untrained hawkers. There is no trained pharmacist in Ndu. Traditional medical care is very common in Ndu and its environs and disease causation, prevention and treatment are always linked to superstition. The “nwe chep” or traditional medicine man is common in this locality.

Ndu is mainly a farming area and most inhabitants are farmers. There are a lot of artificial forests. The climate is made up of two distinct seasons-a long rainy season from March to September and a short dry season from October to February. Temperatures can vary from 17°C in the rainy season around August and during harmattan to about 25°C in the dry season around January. Malaria transmission is seasonal and occurs, mostly, from May to September. Malaria is the main mosquito-borne disease in the locality and as such a public health problem of primary importance. Other diseases common in Ndu are HIV/AIDS, tuberculosis, intestinal helminthes, gastro-enteritis, cough, typhoid, herpes zoster, and skin infections. Cases of filariasis are common but no other mosquito-borne diseases such as yellow fever and elephantiasis have been reported from Ndu to the best of our knowledge. Ndu is predominantly a Christian community. The study took place from July to August 2002.

Ethical approval was obtained from the North West Provincial (now Regional) Delegation of Public Health in Bamenda.

### Study Sample, data collection and analysis

We used a convenient sample of 253 participants. The survey consisted of 112 men and 141 women with different professions (teaching, unskilled labourers, housewives, students, farmers, technicians, nurses, midwives, tea harvesters and cleaners) and age groups. The respondent's consent was sought and gained by explaining the aims of the study. Subjects who could not read or write or understand pidgin English and English were communicated to in “Limbum”, the local language of the Wimbum tribe. A questionnaire was administered to volunteers by trained interviewers according to the time schedule of the participants. Those who could read and/or write answered the questions immediately or at their convenience. The interviewers later collected the filled questionnaires. For those who could not read or write, the interviewers had to meet them at agreed times at home or specific locations to interpret the questionnaire and the participant's responses were recorded. In institutions such as schools, farmer's cooperative societies, group workers like the tea plantation, weekly and monthly contribution meetings known here as “Mngwah”, permission was sought from the leaders after introducing the study and volunteers indicated consent by raising their hands and questionnaires were then administered. In each case, care was taken not to deviate from the closed structure questionnaire. The confidentiality of the information provided was ascertained to the participants. The questionnaire contained independent variables on age, sex, profession and place of abode and questions on antimalarials and dosage, their sources, malaria self-medication and resistance constituted the dependent variables. The chi-square test and confidence intervals were used to compute proportions.

## Results

**Figure 1:**
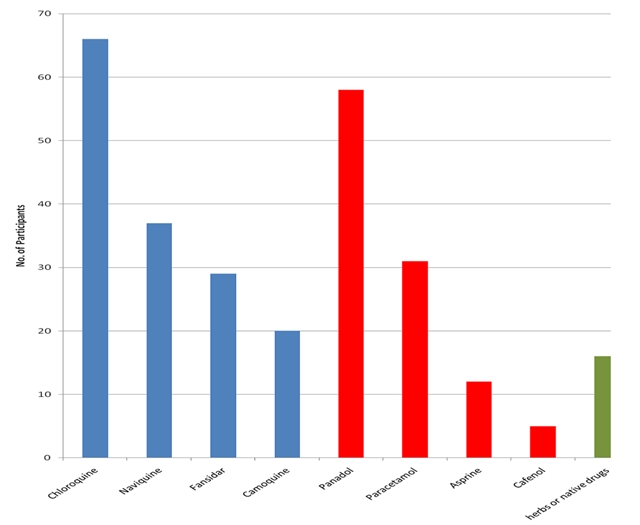
Drugs used for the practice of self-treatment of malaria as stated by the study participants (n=253) Blue: Anti-malarials, Red: Analgesics, Green: Traditional medicne NB: Frequency of responses is greater than 253 because of multiple responses

**Figure 2:**
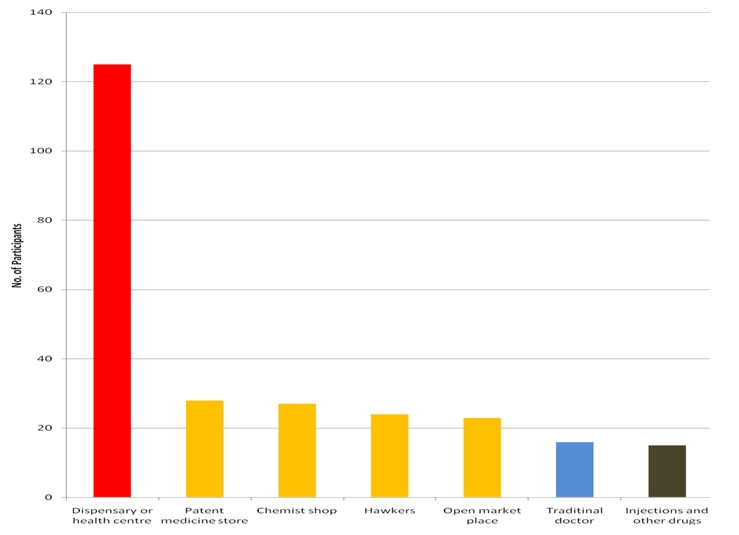
Sources of getting anti-malarials as stated by the study participants (n=253) NB: Frequency of responses is greater than 253 because of multiple responses Red: Medication from authorized sources, Yellow: Medication from unauthorized sources, Blue: Traditional medicine sources, Black: Injections

The demographic characteristics of the study sample are shown in Table 1. Knowledge about the drugs used for the treatment of malaria was very high with 152 (60.1%) participants citing different antimalarial drugs and 106(41.9%) analgesics. The drugs used for the practice of self-treatment (by self-treatment or self-medication, we mean, participants procure and use antimalarials on their own without medical advice or buy them from the roadside drug vendors or quacks) by the participants are shown in Figure 1. 141(55.7%) (95% CI: 49.6–61.8%) participants practiced self-treatment of malaria with medication from various sources while 112 (44.3%) (95% CI: 38–50.6%) did not practice it. More secondary than primary school participants (82 (58.2%) versus 59 (41.8%) respectively) practiced self-treatment when they suspect malaria (P=0.0). The sources of anti-malarials as stated by participants are shown in Figure 2.

**Table 1:** Demographic characteristics of the study participants (n=253)

Demographic characteristics	No (%)
**Age range(years)**	
21–25	24(9.5)
26–30	17(6.7)
31–35	86(34.0)
36–40	21(8.3)
41–45	33(13.0)
46–50	52(20.6)
≥50	20(7.9)
**Sex**	
Male	112(44.3)
Female	141(55.7)
**Occupation**	
Labourers	99(39.1)
Farmers	40(15.8)
Teachers	16(5.3)
Medical personnel	8(3.2)
Housewives	20(7.9)
Students	6(2.4)
Unemployed	64(25.3)
**Tribe**	
Wimbum	228(90.1)
Yambah	09(3.6)
Fulani	11(4.3)
Others	05(2.0)
**Place of abode**	
Hills	116(46.0)
Valleys	137(54.0)
**Educational status**	
Primary and less	155(61.3)
Secondary and above	98(38.7)
**Religion**	
Christian	217(85.8)
Muslim	11(4.2)
Others	25(10.0)

No: Number,%: Percentage

Only 66 (26.1%) (95% CI: 20.7–31.5%) participants knew the correct adult dosage for chloroquine or nivaquine treatment of malaria while 187 (73.9%) did not know. Knowledge of antimalarial drug resistance was poor with 85(33.6%) (95% CI: 27.8–36.6%) participants having the correct knowledge and 168 (66.4%) (95% CI: 60.2–72.2%) having incorrect knowledge. The reasons justifying antimalarial drug resistance as stated by the participants show that there was a statistically significant difference between knowledge of antimalarial drug resistance among the male and female participants as shown in Table 2 (p=0.01). 30(26.8%) (95%CI: 19.0–35.2%) men and 55(39.0%) (95%CI: 31–47%) women gave correct reasons for antimalarial drug resistance. Table 3 shows that knowledge of antimalarial drug resistance was highest among the age group 31–35. No statistically significant relationship was established between correct knowledge of antimalarial drug resistance with age group (p=0.1). From Table 4, most participants who had correct knowledge of antimalarial drug resistance were the unemployed, teachers and labourers. A statistically significant relationship was established between knowledge of antimalarial drug resistance and the profession of the participants (p=0.0).

**Table 2:** Variation of knowledge of reasons of antimalarial drug resistance with gender among the study participants (n=253)

Reasons for malaria resistance	No (%)	Gender	
		
		Male No (%)	Female No (%)
**Correct Reasons**			
When fever continues for a long time during treatment	52(20.6)	15(13.4)	37 (26.2)
When fever is severe during treatment	15(5.9)	8(7.1)	7(5.0)
When chloroquine does not stop the fever	18(7.1)	7(6.3)	11(7.8)
**Incorrect Reasons**			
Don't know	126 (49.8)	70(62.5)	56(39.7)
No response	42(16.6)	12(10.7)	30(12.3)
**Total**	**253(100)**	**112(44.3)**	**141(55.7)**

X^2^=4.6, P-value=0.01; No: Number; %: Percentage

**Table 3:** Variation of knowledge of antimalarial drug resistance among different age groups of the study participants (n=253)

Age group (Years)	Knowledge of malaria resistance		
		
	Know No (%)	Don't know No (%)	Total No (%)
21–25	14(16.5)	10(6.0)	24(9.5)
26–30	6(7.1)	11(6.6)	17(6.7)
31–35	23(27.1)	63(37.5)	86 (34.0)
36–40	8(9.4)	13(7.7)	21(8.3)
41–45	12(14.1)	21(12.5)	33(13.0)
46–50	18(21.2)	34(20.2)	52(20.6)
≥50	4(4.7)	16(5.2)	20(7.9)
**Total**	**85(33.6)**	**168(66.4)**	**253(100)**

X^2^=10.4, df=6, P-value=0.1; No: Number; %: Percentage;

**Table 4:** Variation of knowledge of antimalarial drug resistance and profession of the study participants (n=253)

Profession of participants	Knowledge of malaria resistance		
		
	Know No (%)	Don't know No (%)	Total No (%)
**Professions requiring a certain level of education**			
Medical staff	8(9.4)	0(0.0)	08(3.2)
Teaching	16(18.8)	0(0.0)	16(6.3)
Students	4(4.7)	2(1.2)	06(2.4)
**Professions requiring no level of education**			
Farming	10(11.8)	30(17.9)	40(15.8)
Labourers	14(16.5)	85(50.6)	99(39.1)
Housewives	14(16.5)	6(3.6)	20(7.9)
Unemployed	19(22.4)	45(26.8)	64(25.3)
**Total**	**85(33.6)**	**168(66.4)**	**253(100)**

X^2^=80.8, df=6, p=0.0; No: Number; %: Percentage

## Discussion

There is increasing recognition among health professionals that improving the health of poor people depends upon adequate understanding of the socio-cultural aspects of the context in which public health programmes are implemented [23]. Our study on knowledge and practices is important because it investigated the health seeking behaviour and provides information on health-seeking practices on malaria of the inhabitants of Ndu. The survey also enabled us to understand the culture-specific knowledge on malaria determinants and treatment practices and to relate them to the treatment policy in Cameroon.

### Drugs used for the treatment of malaria

This study took place in 2002 when chloroquine was still the drug of choice for malaria treatment in Cameroon. The most common antimalarial used in Ndu for self-medication was chloroquine even though it has been banned in Cameroon since 2003 because of widespread resistance(in September 2008, the lead author observed hawkers selling chloroquine to patients in Ndop-about 120km from Ndu). In 2005, Cameroon changed the malaria treatment policy from chloroquine or amodiaquine monotherapy to artemisinin-based combination therapy (AS/AQ) [4, 5]. As noted by Sayang and colleagues [4], chloroquine remained available in confessional health facilities in rural areas in Cameroon in 2005. Health education to explore beliefs and to correct misconceptions should precede and accompany the introduction of technological interventions in malaria control programmes in traditional Cameroon societies as noted by Einterz [9]. Drugs used for the treatment of malaria in the study area included antimalarials (chloroquine, nivaquine, fansidar, camoquine), analgesics (panadol, paracetamol, aspirine, cafenol) and native drugs or “Mchep à lah”. Knowledge of the conventional drugs used for the treatment of malaria was very high. This could be due to the strategic location of the town on the border with Nigeria which allows interactions with drug sellers or quack drug dispensers from unauthorized sources. Self-medication has been confirmed in Western Kenya [24] and Dakar, Senegal [25]. This practice may be due to relaxed rules on drug acquisition and can be controlled through stricter rules on anti-malarial drugs procurement. However, self-medication cannot be fully condemned as lives are often saved through this practice. It could be improved upon by educating the local population on the correct use of drugs and their dosages.

### Sources of drugs used for the treatment of malaria

More than 50% of participants got their antimalarials from unauthorized sources which can act as a hurdle to the control of the disease. In 2005, Nkuo Akenji and associates [8] found that proper education of Cameroonian villagers on malaria and presence of health facilities are important strategies that could reduce malaria morbidity and mortality significantly. Most of the drugs used for treating malaria were from unauthorised sources. This agrees with the findings of a Zambian study [26] in isolated rural settings; that the most concerned by malaria prevalence, have difficulties to be educated about treatment progresses and have limited access to official information. Majority of the participants were characterized by low income workers but this did not affect the respondent's source(s) of antimalarials because these drugs are available from many sources including “chemist” shops or medicine stores owned by lay business people who are not trained or licensed pharmacists. In a similar study in Kenya [27] proportions of these responses were much higher. Many reasons could be given for these differences: beliefs that there is no respect of patients at health institutions, cost of acquiring antimalarials and waiting time before being attended to in a health facility [27, 28]. Getting antimalarials from hawkers, unauthorized patent medicine stores, injections and other drugs from unauthorized sources in the “quarters”, the open market and the “Nwe chep” are common in this locality. The practice of consulting the traditional doctor or “Nwe chep” for the causes and prevention of illnesses is common and may explain why some participants patronize traditional doctors. Our findings agree with the work of Good [29] who discovered that traditional ideas may be erroneous from the biomedical perspective and form obstacles to appropriate behaviour and treatment seeking practices.

The presence of many patent medicine stores and other drug sources in the locality could be a motivating factor for the population to get their antimalarials since no medical prescription is needed before drugs are sold. Self-medication can lead to antimalarial drug resistance because there is under- and over-dosing since most drug dispensers are not trained medical personnel. Also, most of the people did not know the correct chloroquine/nivaquine malaria dosage. Self-medication is not limited to this study area; it is common in other areas of Africa [24, 25, 30, 31]. People self-medicate because of the convenience of the readily available drugs they store at home [32], they avoid long distances to the health care provider or lack funds to visit recognized health facilities.

### Variation of knowledge and practice with demographic characteristics of participants

In Africa, malaria affects mainly young children and pregnant women [15, 33] and women have the responsibility for providing nursing and health care for children [34]. Women are therefore more likely to seek and use antimalarials [30] than men, hence their better knowledge of antimalarial drug resistance. Knowledge of antimalarial drug resistance in the study area was low with only 33.6% participants having the correct knowledge. Since self-medication is common in this area, the respondent's knowledge of antimalarial drug resistance was expected to be high. This could be due to the respondents’ experiences from prolonged treatment, severe fever or severe fever during treatment (reasons cited by participants for antimalarial drug resistance), and delays in finding the cause of the illness like consulting the “Nwe chep” or which “god” to appease; hence antimalarial drug resistance is never acknowledged as a problem. Our findings on antimalarial drug resistance agree with the work of Sayang and associates [4] who found persistent fever occurring at day 3 post-treatment (associated or not associated with other clinical signs) as the main criteria to evaluate malaria therapeutic failure in Cameroon.

In professions such as in medicine and teaching where people have attained a certain level of education, there was a statistically significant difference in the knowledge of anti malarial drug resistance between these categories and other professions that don't require any formal educational training. Older people (>31years) may have suffered from many episodes of malarial treatment and tend to know more of antimalarial drug resistance than the younger ones.

## Conclusion

Home treatment or self-medication of malaria is very common and can easily lead to drug resistance in this locality. The best solution to self-medication is that it should be improved upon. To ban it means encouraging people to practice it more in hiding. Improvement in self-treatment of malaria could be effected by providing clear information on choices of drugs for malaria treatment. Proper health information on the rational use of anti-malarial drugs must be provided in an appropriate manner to all groups of people in the society including village health workers, women associations, churches, school children, “Mngwah” opinion leaders, herbalists, health workers and chemists. Advertisements of the correct dosage of common antimalarials on radio, television, posters and newspapers would be good. Training of hawkers and drug store keepers of Ndu on antimalarial dosages and dispensing can also improve the control of the disease.
